# Incidence and risk factors of COVID-19-like symptoms in the French general population during the lockdown period: a multi-cohort study

**DOI:** 10.1186/s12879-021-05864-8

**Published:** 2021-02-10

**Authors:** Fabrice Carrat, Mathilde Touvier, Gianluca Severi, Laurence Meyer, Florence Jusot, Nathanael Lapidus, Delphine Rahib, Nathalie Lydié, Marie-Aline Charles, Pierre-Yves Ancel, Alexandra Rouquette, Xavier de Lamballerie, Marie Zins, Nathalie Bajos, for the SAPRIS study group, Nathalie Bajos, Fabrice Carrat, Pierre-Yves Ancel, Marie-Aline Charles, Florence Jusot, Claude Martin, Laurence Meyer, Ariane Pailhé, Gianluca Severi, Alexis Spire, Mathilde Touvier, Marie Zins

**Affiliations:** 1 Sorbonne Université, Inserm, Institut Pierre-Louis d’Epidémiologie et de Santé Publique, 27 rue Chaligny, 75571 CEDEX 12 Paris, France; 2 Département de Santé Publique, APHP. Sorbonne Université, Paris, France; 3 Sorbonne Paris Nord University, Inserm U1153, Inrae U1125, Cnam, Nutritional Epidemiology Research Team (EREN), Epidemiology and Statistics Research Center – University of Paris (CRESS), Bobigny, France; 4 CESP UMR1018, Université Paris-Saclay, UVSQ, Inserm, Gustave Roussy, Villejuif, Paris, France; 5 Department of Statistics, Computer Science and Applications, University of Florence, Florence, Italy; 6 Université Paris Saclay, Inserm, CESP U1018, Le Kremlin Bicêtre, Paris, France; 7 Service de Santé Publique, APHP. Paris Saclay, Le Kremlin Bicêtre, France; 8 Université Paris-Dauphine, PSL-Research University, LEDa, Paris, France; 9 Santé publique France, Saint-Maurice, France; 10 Ined, Inserm, EFS, UMS Elfe, Aubervilliers, Paris, France; 11 Obstetrical, Perinatal and Pediatric Epidemiology Research Team, Center for Epidemiology and Statistics Sorbonne Paris Cité, INSERM U1153, Paris Descartes University, Paris, France; 12 Clinical Research Unit, Center for Clinical Investigation P1419, Cochin Broca Hôtel-Dieu Hospital, Paris, France; 13 Unité des Virus Emergents, UVE: Aix Marseille Univ, IRD 190, INSERM 1207, IHU Méditerranée Infection, 13005 Marseille, France; 14 Paris University, Paris, France; 15 Paris Saclay University, Inserm UMS 11, Villejuif, France; 16 IRIS, Inserm/EHESS/CNRS, Aubervilliers, France

**Keywords:** COVID-19, General population, Cohort, Incidence, Risk factors, SARS-CoV-2

## Abstract

**Background:**

Our main objectives were to estimate the incidence of illnesses presumably caused by SARS-CoV-2 infection during the lockdown period and to identify the associated risk factors.

**Methods:**

Participants from 3 adult cohorts in the general population in France were invited to participate in a survey on COVID-19. The main outcome was COVID-19-Like Symptoms (CLS), defined as a sudden onset of cough, fever, dyspnea, ageusia and/or anosmia, that lasted more than 3 days and occurred during the 17 days before the survey. We used delayed-entry Cox models to identify associated factors.

**Results:**

Between April 2, 2020 and May 12, 2020, 279,478 participants were invited, 116,903 validated the questionnaire and 106,848 were included in the analysis. Three thousand thirty-five cases of CLS were reported during 62,099 person-months of follow-up. The cumulative incidences of CLS were 6.2% (95% Confidence Interval (95%CI): 5.7%; 6.6%) on day 15 and 8.8% (95%CI 8.3%; 9.2%) on day 45 of lockdown. The risk of CLS was lower in older age groups and higher in French regions with a high prevalence of SARS-CoV-2 infection, in participants living in cities > 100,000 inhabitants (vs rural areas), when at least one child or adolescent was living in the same household, in overweight or obese people, and in people with chronic respiratory diseases, anxiety or depression or chronic diseases other than diabetes, cancer, hypertension or cardiovascular diseases.

**Conclusion:**

The incidence of CLS in the general population remained high during the first 2 weeks of lockdown, and decreased significantly thereafter. Modifiable and non-modifiable risk factors were identified.

**Supplementary Information:**

The online version contains supplementary material available at 10.1186/s12879-021-05864-8.

## Strengths and limitations of this study

- Lockdown was associated with a strong decrease in the incidence of COVID-19-Like Symptoms (CLS) in the French adult population.
- We identified several risk factors of CLS during this period, and we described the immediate consequences in terms of access to healthcare and treatment.
- The most important limitation was the lack of virological confirmation of CLS and the risk of misclassification of a SARS-CoV-2 infection and a disease from another etiology.
- Although participation bias was accounted for with an appropriate weighting method, our findings should not be considered to be strictly representative of the general adult population in France

## Introduction

Following the identification of a novel coronavirus (SARS-CoV-2) in Wuhan, China in December 2019 and its worldwide spread [1], the first imported COVID-19 cases were initially reported in France on January 24, 2020 [2]. Less than 2 months later, the French government declared a nationwide epidemic (phase 3) and a generalized lockdown procedure was set-up on March 17, 2020 [3]. The lockdown included banning of any non-essential public gatherings, closure of educational and public/cultural institutions, ordering people to stay home apart from exercise and essential tasks. Children and their parents were required to stay at home as much as possible [4]. Public health reports have shown that lockdown had a marked impact on the dynamics of the pandemic with a clear downward trend in new hospitalizations from April 1, 2020, and a consecutive decrease in the number of deaths from April 7, 2020 [4, 5]. Thus, the French government eased these restrictions on May 11, 2020 [3]. Although lockdown appeared to successfully alleviate the burden of severe COVID-19 [6], estimates of its impact on mild-to-moderate COVID-19 are based on modelling studies [7], and are not yet supported by clinical evidence.

Our main goals were 1) to estimate the incidence of illnesses presumably caused by SARS-CoV-2 infection during the lockdown period; 2) to identify the associated risk factors. We also described associated symptoms, preventive behaviors and healthcare in relation to these illnesses.

### Participants and methods

#### Design

The SAPRIS (“SAnté, Perception, pratiques, Relations et Inégalités Sociales en population générale pendant la crise COVID-19”) survey was began in March 2020 to evaluate the main epidemiological, social and behavioral challenges of the SARS-CoV2 epidemic in France in relation to social inequalities in health and healthcare. SAPRIS is based on a consortia of prospective cohort studies involving two child-cohorts (not presented in this study) and three general population-based adult cohorts:

- 1) CONSTANCES, a “general population” cohort including 204,973 adults aged 18 to 69 at inclusion and randomly selected from 2012 to be a representative sample of the French adult population affiliated to the General Health Insurance Fund (the source population, that is, approximately 85% of the total French population) [8]. Among CONSTANCES participants, 66,881 are followed by internet, the rest through mailed questionnaires.

- 2) E3N / E4N, a multigenerational adult cohort based on a community of families with 113,000 participants (including women recruited in 1990 and still actively followed-up, their offspring and the fathers of these offspring) among whom 89,606 followed by internet, the rest through mailed questionnaires [9].

- 3) NutriNet-Santé, a nutritional general population-based internet cohort started in 2009, with 170,000 included participants among whom 151,122 were still followed-up in 2020 [10].

*Ethics and public involvement.*

Ethical approval and written informed consent was obtained from each participant before enrolment in the original cohort. According to French law, the present nested survey did not require specific additional written consent from the participant. It was approved by the Inserm ethics evaluation committee (approval #20–672 dated March 30, 2020). Volunteer participants were involved in testing the readability, the comprehension and acceptability of the questions as well as the time required to complete the questionnaires, but they did not contribute to other aspects related to the design, conduct, reporting or dissemination of the research.

**Fig. 1:**
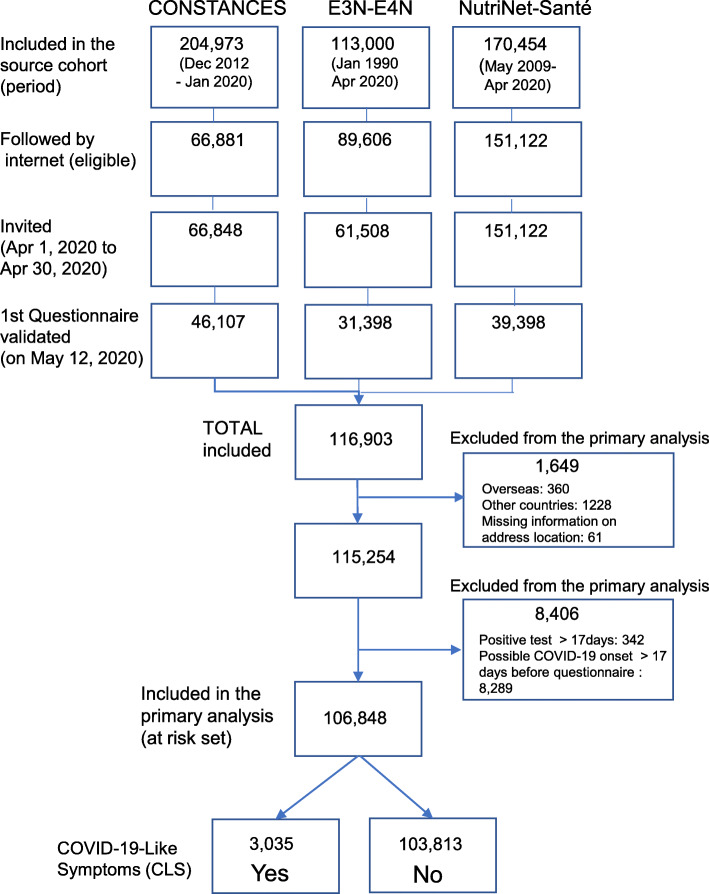
Participants Flow chart

All participants from the original cohorts followed using electronic (internet) questionnaires and who were still under active follow-up on April 1, 2020 (*n* = 279,478) were invited to participate in the current SAPRIS survey (Fig. 1). There were no restrictions on inclusion criteria in the survey. A first self-administered questionnaire covered the lockdown period and was sent from April 1, 2020 and returned before May 12, 2020. A second questionnaire covered the post-lockdown period and was sent between May 5, 2020 and June 15, 2020. The present study used the data from the first self-administered questionnaire, which included questions on socio-demographics, household size and composition, SARS-CoV2 diagnosis, a detailed description of the subject’s symptoms in the 2 weeks before the questionnaire, comorbidities, healthcare use and treatment, employment, daily life, child care, alcohol, tobacco and cannabis use, social and sexual life, preventive measures, risk perception and beliefs.

Additional specific socio-demographic and clinical characteristics were extracted from original cohort databases. Symptoms were reported if they had been present at least once in the last 14 days. If a symptom had been, but was no longer present when the questionnaire was completed, the duration was noted on a scale (less than 1 day, one to 3 days, four to 7 days, eight to 14 days, > 14 days). Finally, the total time (in days) between the onset of the first symptoms and the questionnaire was reported. All visits outside the home and the use of preventive measures in the 7 days before the questionnaire were reported.

#### Outcome

The main outcome was COVID-19-Like symptoms (CLS), defined according to the European Centre for Disease Prevention and Control as at least one of a cough, a fever, a dyspnea, a sudden onset of anosmia, ageusia or dysgeusia [11], that lasted more than 3 days and occurred during the at-risk period. Participants were also requested to report the occurrence of cough, fever or dyspnea before March 1, 2020 or between March 1 and the 2 weeks before the questionnaire, and whether they or any other household members had tested positive for SARS-CoV-2 before the questionnaire. The primary “at-risk period” was defined as the 17 days before the self- administered questionnaire for each patient, corresponding to the 14 days to report the presence of symptoms, plus 3 days for the minimum duration of our definition of CLS. In a first sensitivity analysis, no restriction was made on the minimum duration of symptoms, extending our primary case-definition of CLS to illness that lasted less than 4 days. In a second sensitivity analysis, the at-risk period was defined as between March 16, 2020 and the date of the questionnaire for all participants. This definition made it possible to include all CLS that occurred during the lockdown period.

#### Covariates

We explored the association of CLS with age, gender, address location, French metropolitan regions, number of people living in the household, number of children in the household**,** educational level, professional activity before lockdown, job position, professional activity during lockdown, BMI, chronic conditions (according to a pre-specified list). Age groups were categorized according to predefined limits (< 40 (reference level); 40–49; 50–59; 60–69; ≥70 years) and BMI according to standard cut-offs (< 18.5; 18.5- < 25 (reference level); ≥25- < 30 (overweight); ≥30 (obese) kg/m^2^) [12].

#### Statistical methods

We determined that 100,000 subjects were needed to have a power of at least 80% to identify associations (Odds-Ratio, OR < 0.9 or OR > 1.1) between covariates and CLS in a wide range of situations, assuming 10% of events and 10 to 90% exposure.

We used inverse probability weighting to correct for selection bias (when only a subgroup of the whole cohort was invited to participate by internet) and inverse probability weighting to correct for non-participation bias in those who were invited. Weights were estimated using logistic regression models, with selection or participation as the response variables, and participants’ characteristics as covariates (see supplementary Table 1). Unweighted and weighted daily incidence rates of CLS and 95% confidence intervals were estimated with an exact method based on the Poisson distribution. Estimates of unweighted and weighted cumulative incidences on days 15 (March 30, 2020), 30 (April 14, 2020) and 45 (April 29, 2020) of lockdown were obtained as one minus the estimated probability of survival free of CLS at that time.

To account for potential heterogeneity between the cohorts, left-truncation and censorship in the data, factors associated with the occurrence of CLS were identified using unweighted data and delayed-entry Cox models with stratification on the source cohort. The start of the at-risk period was defined according to the calendar day for each participant and survival time was calculated as the time between that day and the day the questionnaire was filled-out in case of no symptom or the day the first symptoms were reported in CLS cases. Multivariable analysis was performed including all factors associated with CLS cases on univariable analysis. All analyses were performed with SAS 9·4 software (SAS Institute Inc., Cary, North Carolina, USA). A *P*-value <.05 was considered to be statistically significant.

## Results

A total of 116,903 of the 279,478 participants (42%) who were invited to participate in the survey completed the questionnaire. The participation rate was 69% in the CONSTANCES cohort, 51% in the E3NE4N cohorts and 26% in the NutriNet-Santé cohort (Fig. 1).

Table 1 presents the characteristics of included participants. Median age was 59 years old (Q1-Q3: 46 to 71 years), and 66% of the participants were women. Twenty-six percent were residents of the Ile-de-France or GrandEst regions – the two regions with the highest rate of SARS-CoV-2 in metropolitan France, while 23% lived in rural areas and 44% lived in cities of more than 100,000 inhabitants. At least one child or adolescent was present at home in 25%. Forty-three percent were retired and 50% were working adults, but only 8% worked outside the home during the lockdown period. Ten percent of the participants were obese and a chronic disease was reported in 34% of participants.

**Table 1:** Participants’ characteristics

	Constances *N* = 46,107 (39)^a^	E3NE4N *N* = 31,398 (27)			NutriNet-Santé *N* = 39,398 (34)	Total adults 116,903 (100)
		E3N *N* = 16,744	E4NG1 *N* = 4865	E4NG2 *N* = 9789		
Questionnaires						
First	Apr 6, 2020	Apr 17, 2020	Apr 17, 2020	Apr 9, 2020	Apr 1, 2020	Apr 1, 2020
Last	May 5, 2020	May 8, 2020	May 7, 2020	May 6, 2020	May 12, 2020	May 12, 2020
Age group (years)						
< 40	9504 (21)^a^	0 (0)	0 (0)	764 (8)	6296 (16)	16,564 (14)
[40–50[	10,483 (23)	0 (0)	0 (0)	4016 (41)	6701 (17)	21,200 (18)
[50–60[	9400 (20)	0 (0)	3 (1)	3820 (39)	7799 (20)	21,022 (18)
[60–70[	11,408 (25)	0 (0)	273 (6)	1146 (12)	9933 (25)	22,760 (19)
> =70	5312 (16)	16,744 (100)	4589 (94)	43 (0)	8669 (22)	35,357 (30)
Gender						
Female	23,426 (51)	16,744 (100)	0 (0)	6483 (66)	30,130 (76)	76,783 (66)
Male	22,681 (49)	0 (0)	4865 (100)	3306 (34)	9268 (34)	40,120 (34)
Regions						
Ile-de-France	7856 (17)	2760 (16)	707 (15)	2440 (25)	7280 (18)	21,043 (18)
Grand-Est	4165 (9)	1308 (8)	387 (8)	615 (6)	2903 (7)	9378 (8)
Other French metropolitan regions	34,086 (74)	12,673 (76)	3770 (77)	6218 (64)	28,086 (71)	84,833 (73)
French Overseas	0 (0)	0 (0)	1 (0)	54 (1)	305 (1)	360 (0)
Foreign countries	0 (0)	3 (0)	0 (0)	401 (4)	824 (2)	1228 (1)
Missing	0	0	0	61	0	61
Living Area						
Rural	3349 (8)	5125 (31)	1613 (33)	2784 (29)	13,550 (34)	26,421 (23)
< 20,000 inhab.	1310 (3)	4526 (27)	1454 (30)	2330 (24)	9174 (23)	18,794 (16)
20–000-100,000 inhab.	2871 (7)	3989 (24)	1061 (22)	2254 (23)	8886 (23)	19,061 (17)
> 100,000 inhab.	36,473 (83)	3005 (18)	722 (15)	2389 (24)	7788 (20)	50,377 (44)
Missing	2104	99	15	32	0	2250
Household size and composition						
Nb persons (incl. participant)						
1	6461 (14)	4869 (30)	168 (4)	1200 (12)	7855 (20)	20,553 (18)
2	18,963 (41)	10,601 (65)	4260 (91)	2465 (25)	18,032 (46)	54,231 (47)
3–6	20,274 (44)	751 (5)	253 (5)	5990 (61)	13,360 (34)	40,268 (35)
7 or +	248 (1)	44 (0)	6 (0)	87 (1)	151 (0)	536 (0)
Missing	161	479	178	47	0	865
Nb children (< 18 yrs)						
0	31,462 (68)	16,089 (99)	4640 (99)	5033 (52)	29,841 (76)	87,908 (75)
1	5637 (12)	100 (1)	33 (1)	1852 (19)	4063 (10)	11,693 (10)
2	6761 (15)	61 (0)	11 (0)	2151 (22)	4189 (11)	13,186 (11)
3–6	2078 (5)	13 (0)	3 (0)	701 (7)	1301 (3)	4097 (4)
7 or +	8 (0)	2 (0)	0 (0)	5 (0)	4 (0)	19 (0)
Missing	161	479	178	47	0	865
Educational level						
< High-school degree	5843 (13)	971 (6)	595 (12)		5830 (15)	13,239 (13)
High-school degree or undergraduate	23,839 (53)	7486 (46)	1335 (28)		17,247 (44)	49,907 (47)
Graduate degree or doctorate	15,257 (34)	7790 (48)	2906 (60)		16,060 (41)	42,013 (40)
Other or Missing	1168	497	29	9789	261	11,744
Professional activity before lockdown						
Student	402 (1)	6 (0)	0 (0)	19 (0)	426 (1)	853 (1)
Working	29,153 (63)	47 (0)	31 (1)	8299 (85)	20,529 (52)	58,059 (50)
Looking for a job	1543 (3)	1 (0)	3 (0)	336 (3)	1128 (3)	3111 (3)
Retired	13,678 (30)	16,188 (97)	4705 (97)	756 (8)	15,368 (39)	50,695 (43)
Not working due to health conditions	383 (1)	9 (0)	3 (0)	110 (1)	552 (1)	1057 (1)
No professional activity (housewife or husband)	806 (2)	438 (3)	113 (2)	205 (2)	1295 (3)	2857 (2)
Missing	142	55	10	64	0	271
Essential job position						
Healthcare worker	1968 (4)	0 (0)	1 (0)	555 (6)	1744 (4)	4268 (4)
Other essential job	5330 (12)	6 (0)	2 (0)	1423 (15)	4250 (11)	11,011 (9)
Professional activity during lockdown						
Not working	16,812 (36)	16,642 (100)	4824 (100)	1426 (16)	18,869 (53)	58,873 (54)
Stopped working	2304 (5)		0 (0)	423 (5)	1703 (5)	4442 (4)
Working from home, remote working	15,030 (35)	12 (0)	14 (0)	5015 (56)	8910 (25)	28,986 (27)
Partially working from home	2908 (7)	17 (0)	5 (0)	899 (10)	2201 (6)	6015 (6)
Working outside home	4189 (10)	2 (0)	1 (0)	902 (10)	3614 (10)	8707 (8)
Other	1321 (3)	1 (0)	3 (0)	359 (4)	104 (0)	1789 (2)
Missing	3543	2 (0) 68	18	765	3997	8391
BMI (kg/m^2^)						
< 18.5	1147 (2)	633 (4)	31 (1)	296 (4)	1752 (5)	3859 (3)
[18.5; 25[	26,254 (58)	9621 (59)	2385 (49)	5173 (63)	23,054 (60)	66,487 (59)
[25; 30[(overweight)	13,320 (30)	4597 (28)	2023 (42)	1944 (24)	9538 (25)	31,422 (28)
> =30 (obese)	4173 (9)	1402 (9)	382 (8)	763 (9)	4098 (11)	10,818 (10)
Missing	1213	491	44	1613	956	3873
Chronic diseases						
Yes	11,777 (26)	7984 (48)	2826 (58)	2338 (24)	14,310 (36)	39,235 (34)
No	33,891 (74)	8490 (51)	1977 (41)	7378 (75)	24,752 (21)	76,488 (66)
Don’t know	284 (1)	195 (1)	51 (1)	65 (1)	336 (1)	931 (1)
Missing	155	75	11	8	0	249
Chronic diseases						
Asthma, COPD, other respir. Diseases	1399 (3)	5794 (35)	1572 (32)	295 (3)	7042 (18)	16,102 (14)
Diabetes	690 (2)	3252 (20)	1127 (23)	127 (1)	1417 (4)	6613 (6)
Hypertension	2993 (7)	3275 (20)	1218 (25)	469 (5)	4787 (12)	12,742 (11)
Other cardiovascular diseases	934 (2)	1012 (6)	727 (15)	101 (1)	1293 (3)	4067 (3)
Cancer	602 (1)	755 (5)	426 (9)	108 (1)	4525 (11)	6416 (6)
Anxiety, depression	1083 (2)	619 (4)	106 (2)	267 (3)	1323 (3)	3398 (3)
Other	3253 (7)	3430 (21)	791 (16)	569 (6)	7408 (19)	15,451 (13)
Missing	142	55	10	64	0	249

^a^ n (%)

Participants who were living outside mainland France (*n* = 1588) or with missing information on their exact address location (*n* = 61) or who reported a positive SARS-CoV-2 test result (*n* = 342) and/or a CLS onset (*n* = 8289) before the at-risk period were excluded from the primary analysis. The primary analysis evaluated 106,848 participants: 3035 CLS were reported during 62,099 person-months of follow-up. The unweighted cumulative incidences of CLS were 6.2% (95% Confidence Interval (95%CI): 5.7%; 6.6%), 7.7% (95%CI 7.3%; 8.2%) and 8.8% (95%CI 8.3%; 9.2%) on days 15, 30 and 45 of lockdown, respectively. The weighted cumulative incidences were 7.2% (95%CI 6.7%; 7.8%), 9.0% (95%CI 8.4%; 9.5%) and 10.1% (95%CI 9.6%; 10.6%) on days 15, 30 and 45 of lockdown, respectively.

Extending CLS definition to illness that lasted less than 4 days, 5313 cases were identified during 59,768 person-months of follow-up with unweighted cumulative incidences of 9.7% (95%CI 9.2%; 10.3%), 12.6% (95%CI 12.0%; 13.1%) and 14.3% (95%CI 13.7%;14.9%) on days 15, 30 and 45 of lockdown, respectively. Sensitivity analysis of all cases of CLS onset after March 16, 2020 excluded 171 participants with a positive test result and/or 4084 with the onset of CLS before March 16, 2020 and identified 7240 cases in 110,207 person-months of follow-up with unweighted cumulative incidences of 4.7% (95%CI 4.5%; 4.8%), 6.6% (95%CI 6.5%; 6.8%) and 7.7% (95%CI 7.5%;7.9%) on days 15, 30 and 45 of lockdown, respectively.

**Fig. 2:**
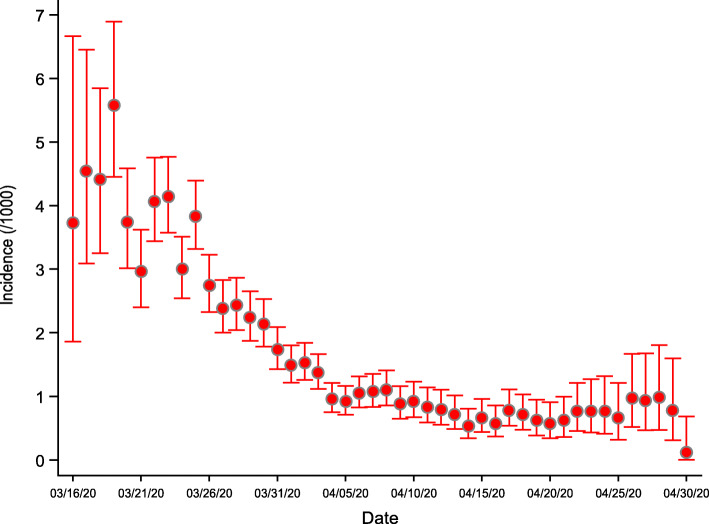
Daily incidence (/1000) of COVID-19-Like Symptoms (> 3 days) in participants between March 16, 2020 (first day of the lockdown in France) and April 30, 2020 - unweighted estimates. Error bars indicate 95% confidence intervals

The primary daily incidence rate peaked on day four of lockdown (March 19, 2020; unweighted estimate 5.57 per 1000 person-days (95%CI 4.45; 6.89) – Fig. 2) and showed a sharp and constant decrease to reach less than 1 per 1000 person-days after day 25 (April 9, 2020). Similar findings were observed in the weighted incidence rates and the sensitivity analysis considering a different at-risk period (supplementary Figs. 1&2). Daily incidence rates were higher but showed a similar temporal pattern when the case-definition of CLS included illness that lasted less than 4 days (supplementary Fig. 3).

Eighty out of 189 participants who experienced CLS and were tested reported a positive (RT-PCR) test result (supplementary Table 2). Headaches, rhinorrhea and fatigue were frequently reported in addition to the symptoms defining CLS. Eight hundred and forty-eight (28%) participants with CLS had a GP or a hospital visit, and a diagnosis of COVID-19 was considered to be very likely or likely by the physician in 62% cases. Paracetamol was taken by 62% and antibiotics by 6% of participants with CLS. Only 8 participants used chloroquine or hydroxychloroquine. Forty percent participants stayed strictly confined at home following symptoms onset.

Table 2 presents the unweighted incidence rates of CLS and the hazard ratios obtained from the univariable Cox models with stratification on source cohort. Almost all tested factors were found to be associated with CLS. A positive RT-PCR in another household member was strongly associated with CLS in the participant; this variable was not included in the multivariable analysis to avoid overfitting. On multivariable analysis (Table 3), the risk of COVID-19 was lower in older age groups and was higher in the Ile-de-France and GrandEst regions (compared to other French metropolitan regions), in those living in cities > 100,000 inhabitants (vs rural areas), when at least one child or adolescent was living in the same household, in overweight or obese participants, and in people with chronic respiratory diseases, anxiety or depression and chronic diseases other than diabetes, cancer, hypertension or other cardiovascular diseases. The observed associations were confirmed in the sensitivity analyses, except that male gender, living in a household of size 2 and being retired were negatively associated with the risk of CLS in addition to factors identified in the primary analysis (supplementary Tables 3 & 4).

**Table 2:** Unweighted incidence rates of COVID-19-Like Symptoms (CLS) by covariate values and univariable hazard-ratios^a^

	Number CLS /number person-months	Incidence rate (95%CI) (/100 person-months)	Hazard-Ratio	*P*-Value
Age group (years)				
< 40	732/8561	8.55 (7.94; 9.19)	Reference	
[40–50[	783/10,980	7.13 (6.64; 7.65)	0.87 (0.78; 0.96)	0.0053
[50–60[	619/10,912	5.67 (5.23; 6.14)	0.68 (0.61;0.75)	< 0.0001
[60–70[	480/12,120	3.96 (3.61; 4.33)	0.43 (0.38;0.48)	< 0.0001
> =70	421/19,526	2.16 (1.96; 2.37)	0.27 (0.23; 0.31)	< 0.0001
Gender				
Female	1937/40,626	4.77 (4.56;4.99)	reference	
Male	1098/21,473	5.11 (4.82; 5.43)	0.88 (0.81; 0.95)	0.0011
Regions				
Ile-de-France	697/11,081	6.29 (5.83; 6.78)	1.38 (1.27; 1.51)	< 0.0001
GrandEst	286/4908	5.83 (5.17; 6.54)	1.30 (1.15; 1.48)	< 0.0001
Other French metropolitan regions	2052/46,110	4.45 (4.26; 4.65)	reference	
Living Area				
Rural	539/14,249	3.78 (3.47; 4.12)	Reference	
< 20,000 inhab.	386/10,069	3.83 (3.46; 4.24)	1.07 (0.94; 1.22)	0.2903
20–000-100,000	465/10,114	4.60 (4.19; 5.04)	1.23 (1.09; 1.40)	0.0010
> 100,000 inhab.	1581/26,458	5.98 (5.68; 6.28)	1.26 (1.12; 1.41)	< 0.0001
Missing	64/1209	5.29 (4.08; 6.76)		
Household size and composition				
Nb persons (incl. participant)				
1	495/10,881	4.55 (4.16; 4.97)	Reference	
2	1076/29,241	3.68 (3.46; 3.91)	0.81 (0.73; 0.91)	0.0002
3 or +	1453/21,496	6.76 (6.42; 7.12)	1.36 (1.23; 1.51)	< 0.0001
Missing	11/481	2.29 (1.14; 4.09)		
Nb children (< 18 yrs)				
0	1881/46,600	4.04 (3.86; 4.22)	Reference	< 0.0001
1 or +	1143/15,018	7.61 (7.18; 8.07)	1.77 (1.64; 1.91)	
Missing	11/481	2.29 (1.14; 4.09)		
Educational level				
< High-school degree	275/7178	3.83 (3.39; 4.31)	Reference	
High-school degree or undergraduate	1311/26,739	4.90 (4.64; 5.18)	1.35 (1.19; 1.54)	< 0.0001
Graduate degree or doctorate	1147/22,155	5.18 (4.88; 5.49)	1.56 (1.37; 1.78)	< 0.0001
Missing	302/6027	5.01 (4.46; 5.61)		
Professional activity before lockdown				
Student	36/446	8.07 (5.65; 11.2)	1.18 (0.85; 1.64)	0.3221
Working	2025/30,170	6.71 (6.42; 7.01)	Reference	
Looking for a job	113/1578	7.16 (5.90; 8.61)	1.01 (0.84; 1.22)	0.8955
Retired	740/27,739	2.67 (2.48; 2.87)	0.43 (0.39; 0.47)	< 0.0001
Not working due to health conditions	48/529	9.07 (6.69; 12.0)	1.35 (1.01; 1.79)	0.0415
No professional activity (housewife or husband)	62/1490	4.16 (3.19; 5.33)	0.64 (0.50; 0.83)	0.0006
Missing	11/147	7.50 (3.74; 13.4)		
Essential job position				
Healthcare worker (Y vs N)	161/2207	7.29 (6.21; 8.51)	1.44 (1.23; 1.69)	< 0.0001
Other essential job (Y vs N)	361/5750	6.28 (5.65; 6.96)	1.20 (1.08; 1.35)	0.0010
Professional activity during lockdown				
Not working	999/31,782	3.14 (2.95; 3.34)	0.56 (0.51; 0.62)	< 0.0001
Stopped working	173/2321	7.45 (6.38; 8.65)	1.12 (0.95; 1.32)	0.1691
Working from home, remote working	959/15,052	6.37 (5.97; 6.79)	Reference	
Partially working from home	188/3143	5.98 (5.16; 6.90)	0.97 (0.83; 1.14)	0.7257
Working outside home	313/4573	6.84 (6.11; 7.65)	1.12 (0.99; 1.27)	0.0835
Other	68/951	7.15 (5.55; 9.07)	1.07 (0.83; 1.36)	0.6136
Missing	335/4277	7.83 (7.02; 8.72)		
BMI (kg/m^2^)				
< 18.5	104/2035	5.11 (4.18; 6.19)	1.15 (0.94; 1.40)	0.1746
[18.5; 25[	1621/35,552	4.56 (4.34; 4.79)	Reference	
[25; 30[(overweight)	826/16,650	4.96 (4.63; 5.31)	1.08 (0.99; 1.17)	0.0763
> =30 (obese)	365/5615	6.50 (5.85; 7.20)	1.40 (1.25; 1.56)	< 0.0001
Missing	119/2247	5.30 (4.39; 6.34)		
Chronic diseases				
Yes	1005/20,670	4.86 (4.57; 5.17)	1.12 (1.03; 1.21)	0.0053
No	1993/40,834	4.88 (4.67; 5.10)	Reference	
Didn’t know	34/461	7.38 (5.11; 10.3)	1.73 (1.23; 2.43)	0.0015
Missing	3/135	2.23 (0.46; 6.51)		
Chronic diseases (Y vs N)				
Asthma, COPD, other resp. diseases	415/8319	4.99 (4.52; 5.49)	1.51 (1.35; 1.69)	< 0.0001
Diabetes	113/3582	3.15 (2.60; 3.79)	1.00 (0.83; 1.22)	0.9662
Hypertension	270/6780	3.98 (3.52; 4.49)	0.92 (0.81; 1.04)	0.1682
Other cardiovascular diseases	79/2194	3.60 (2.85; 4.49)	0.88 (0.70; 1.10)	0.2493
Cancer	168/3286	5.11 (4.37; 5.95)	1.14 (0.97; 1.34)	0.1078
Anxiety, depression	132/1702	7.75 (6.49; 9.20)	1.72 (1.44; 2.04)	< 0.0001
Other	432/8003	5.40 (4.90; 5.93)	1.27 (1.15; 1.41)	< 0.0001
Missing	3/135	2.23 (0.46;6.51)		
Positive RT-PCR in another household member				
(Y vs N)	58/207	28.0 (21.2; 36.1)	5.68 (4.38; 7.37)	< 0.0001

^a^ with stratification on cohort study

**Table 3:** Multivariable-adjusted hazard-ratios of COVID-19-Like Symptoms (CLS) according to covariate values^a^

	Hazard-Ratio	*P*-Value
Age group		
< 40	Reference	
[40–50[	0.80 (0.72; 0.90)	< 0.0001
[50–60[	0.68 (0.60;0.76)	< 0.0001
[60–70[	0.52 (0.44;0.62)	< 0.0001
> =70	0.34 (0.27; 0.44)	< 0.0001
Gender		
Female	reference	
Male	0.99 (0.91; 1.07)	0.7267
Regions		
Ile-de-France	1.31 (1.19; 1.44)	< 0.0001
GrandEst	1.29 (1.14; 1.47)	< 0.0001
Other French metropolitan regions	reference	
Living Area		
Rural	Reference	
< 20,000 inhab.	1.07 (0.93; 1.22)	0.3629
20–000-100,000 inhab.	1.14 (1.00; 1.30)	0.0471
> 100,000 inhab.	1.15 (1.02; 1.29)	0.0241
Household size and composition		
Nb persons (incl. participant)		
1	Reference	
2	0.95 (0.85; 1.06)	0.3405
3 or +	1.00 (0.87; 1.16)	0.9603
Nb children (< 18 yrs)		
0	Reference	
1 or +	1.28 (1.13; 1.45)	0.0001
BMI (kg/m2)		
< 18.5	1.04 (0.85; 1.27)	0.6916
[18.5; 25[	Reference	
[25; 30[(overweight)	1.17 (1.07; 1.27)	0.0005
> =30 (obese)	1.41 (1.25; 1.58)	< 0.0001
Professional activity before lockdown		
Student	1.01 (0.71; 1.44)	0.9620
Working	Reference	
Looking for a job	1.05 (0.86; 1.27)	0.6618
Retired	0.86 (0.72; 1.03)	0.1057
Not working due to health conditions	1.30 (0.96; 1.77)	0.0894
No professional activity (house wife or husband)	0.89 (0.68; 1.16)	0.3773
Essential job position		
Health care worker (Y vs N)	1.18 (1.00; 1.40)	0.0535
Other essential job (Y vs N)	1.01 (0.90; 1.14)	0.8922
Chronic diseases (Y vs N)		
Asthma, COPD, other resp. diseases	1.41 (1.24; 1.60)	< 0.0001
Anxiety, depression	1.31 (1.08; 1.58)	0.0065
Other	1.22 (1.08; 1.37)	0.0013

^a^ with stratification on the cohort. 6567 (6%) participants excluded from the multivariable model due to missing values including 190 with CLS; educational level was not significantly associated with CLS when included in the multivariable model (high school degree or undergraduate versus <high-school degree, *P* = 0.7743; graduate or doctorate versus <high-school degree, *P* = 0.6299), and was not kept in the final model due to missing information on this covariate in the E4NG2 cohort. To avoid overfitting Positive RT-PCR in another household member was not entered in the model. However, results were unchanged when this variable was entered (not shown)

## Discussion

Lockdown was associated with a strong decrease in the incidence of CLS in the French adult population that participated in this survey. This study shows that the cumulative incidence of CLS on day 45 of lockdown ranged from 7.7 to 10.2% depending on the estimation method, that more than 60 % of new cases occurred within the first 2 weeks, and that the daily incidence remained at a sustained low level 1 month after lockdown and thereafter. In addition, we identified several risk factors of CLS during this period, and have described the immediate consequences in terms of access to healthcare and treatment associated with these syndromes. To our knowledge this is the first study to report the signs and symptoms of COVID-19 on a nationwide scale and during lockdown.

Only 28% of the participants with CLS had a medical visit. This result is in line with estimates based on a digital participatory system in France during the same period, in which 31% of COVID-19 patients sought medical advice [13]. Forty percent of the participants with symptoms remained strictly confined without leaving their homes, following the government’s recommendations. This low proportion can be explained in part by the need for some participant to go out for basic necessities, for example if the participant lived alone.

Considering the estimated 5-day median incubation time of COVID-19 and the appearance of symptoms within twelve days after infection [14], a large proportion of participants who developed CLS in the first two weeks were probably infected before lockdown, most of them in the community or at the workplace. It is therefore not surprising to find the association of CLS in adults with decreasing age [15], living in urban versus rural environments [16], in highly prevalent French regions [17], all factors that were reported in other studies performed before lockdown.

A lower infection rate with increasing age was reported in several population-based serological studies [18] which is consistent with our findings, although the risk of severe illness or deaths exponentially increases with age among those infected [19]. As in other studies, univariable analysis identified the size of the household (including children), but only living with at least one child or adolescent remained associated with CLS on multivariable analysis, indicating that this age group could play an important role in household-related transmission [20]. We also identified other factors indicating potential secondary household-related transmission, such as living with another person with a positive diagnosis of SARS-CoV-2 [21]. However, it was impossible to determine a timeframe for this factor and identify whether the participant was the source of infection or was infected by a household member. Being a healthcare worker was associated with CLS in univariable analysis - as reported other studies [22, 23], but the association did not remain significant in the multivariable model, potentially due to a lack of statistical power. Obesity has been found to be linked with the risk of severe CLS in young patients [24], and also suspected to increase the susceptibility to infection [25]. Different theories suggest that asthma, COPD and other respiratory diseases may be negatively or positively associated with the susceptibility to SARS-CoV-2 infection due to up or down regulation of angiotensin-converting enzyme-2 expression. However, all of these respiratory diseases have been shown to be associated with the severity of illness in infected persons [26–28]. Since 30 to 60% of SARS-CoV-2 infections are asymptomatic [29–32] and were not included in our CLS cases, by definition, it is not surprising to find the presence of these conditions, which are known to be associated with more severe disease, in subjects with symptomatic SARS-CoV-2 infection. Finally, we found a strong association between CLS and anxiety or depression, which may be related either to a direct (i.e. causal) impact of these comorbidities on the risk of CLS, or to an over-reporting of CLS caused by increased anxiety or stress in this specific subgroup. Although psychiatric disorders have been reported during the acute phase of the infection [33], the risk of reverse causality explaining this association should however be limited as co-morbidities were collected prior to the survey.

Consistent results were obtained in the sensitivity analyses. An association with CLS was found with being retired compared with being working, but the strength of the association was of the same magnitude than estimated in the main analysis. This result can be the consequence of a higher power of the sensitivity analyses due to a higher number of events, and explained by a lower rate of social contacts in this category of persons compared with working people of the same age.

Our study has several limitations. The most important limitation is the lack of virological confirmation of CLS and the risk of misclassification of a SARS-CoV-2 infection and a disease from another etiology. During lockdown, French health authority recommendations limited SARS-CoV-2 testing with a RT-PCR test to patients with severe symptoms requiring hospitalization or to specific situations (e.g. healthcare workers with symptoms). Thus, testing was not available to most participants. Nevertheless, the influenza season peaked on week 6 and ended on week 10 to 12, just before lockdown, which limits the risk of acute respiratory infection caused by an influenza virus. In addition, 42% of the small group of participants who were tested for SARS-CoV-2 infection in our study reported a positive RT-PCR result. This positive rate was higher than that reported in the community (30% at its highest between March 23 and March 29, 2020) [34]. However, a 15 to 20% seroprevalence of SARS-CoV-2 was reported in Spain in individuals from the general population who presented symptoms compatible with COVID-19 [32]. It is therefore likely that the cause of illness was not SARS-CoV-2 infection in a significant proportion of our CLS cases and only studies using sensitive and specific virological methods can accurately quantify the extent of the SARS-CoV-2 epidemic. To avoid recall bias, which is another potential limitation of our study, we limited the questionnaire to the symptoms present in the past 14 days. To avoid a selection bias induced by different dates for filling in the questionnaires resulting in dates of ‘at-risk period’ that varied from one subject to another, we used a Cox model with delayed entry. Finally, although participation bias was accounted for with an appropriate weighting method, our findings should not be considered to be strictly representative of the general adult population in France. Nevertheless, the large number of subjects from all social categories allows us to draw robust conclusions on the factors associated with the occurrence of CLS in France.

## Conclusion

In conclusion, to our knowledge this is the first study to quantify the incidence of CLS in the general population on a nationwide scale and during a lockdown, and it shows the significant impact of lockdown on the dynamics of the incidence of infection. A follow-up study is ongoing and will be combined with SARS-CoV-2 serological tests of all participants to estimate the seroprevalence and identify the associated factors.

## Supplementary Information

**Additional file 1.**

## Data Availability

In regards to data availability, data of the study are protected under the protection of health data regulation set by the French National Commission on Informatics and Liberty (Commission Nationale de l’Informatique et des Libertés, CNIL). The data can be available upon reasonable request to the corresponding author (fabrice.carrat@iplesp.upmc.fr), after a consultation with the steering committee of the Sapris study. The French law forbids us to provide free access to Sapris data; access could however be given by the steering committee after legal verification of the use of the data.

## Abbreviations

OR: Odds-Ratio
CI: Confidence Intervals
