# Supplementary material for: Incidence and risk factors of COVID-19-like symptoms in the French general population during the lockdown period: a multi-cohort study
Source: BMC Infect Dis. 2021 Feb 10;21:169. doi: 10.1186/s12879-021-05864-8 (PMC7875161; doi:10.1186/s12879-021-05864-8)
Supplement: Supplementary file 1 — Additional file 1. [file 12879_2021_5864_MOESM1_ESM.docx]

Supplementary table 1. Covariates used in logistic regression models to estimate selection and participation weights by source cohort.

|  | CONSTANCES | E3NE4N | NutriNet-Santé |
| --- | --- | --- | --- |
| Age group | X | X | X |
| Gender | X | X | X |
| Regions | X | X | X |
| Socio-professional category | X | X | X |
| Size of the household |  | X | X |
| Educational level | X |  |  |
| BMI | X | X |  |
| Perceived health | X | X |  |
| Smoking (status | X | X | X |
| Alcohol consumption | X |  |  |
| Hypercholesterolemia |  | X |  |
| Hypertension | X | X |  |
| Diabetes | X | X |  |
| Asthma | X |  |  |
| Cancer | X |  |  |
| Disability | X |  |  |
| Satisfaction with life lived | X |  |  |
| Confident in her/his financial situation | X |  |  |
| Living in rural or urban environment | X |  |  |
| Type of housing |  | X |  |
| Area deprivation index |  | X |  |

Supplementary table 2. Details of symptoms and outcomes in 3035 participants with COVID-19-Like Symptoms

| Primary endpoint: at least one of cough, fever, dyspnea, sudden onset of anosmia, ageusia or dysgeusia > 3 days with onset in the last 17 days before the self-administered questionnaire | N=3035* |
| --- | --- |
| RT-PCR  Positive  Negative RT-PCR  Untested | 80 (3)  109 (4)  2846 (94) |
| Symptoms  Fever or feverishness  Cough  Dyspnea  Anosmia/ageusia  Headaches  Rhinorrhea  Fatigue  Stiffness, myalgia  Nausea  Diarrhea  Chest pain | 1140 (38)  2144 (71)  839 (28)  515 (17)  1560 (51)  1391 (46)  1367 (45)  993 (33)  340 (11)  675 (22)  755 (25) |
| Personal feeling “I had Covid-19”  Yes  No  Missing | 1766 (58)  1258 (42)  11 |
| Immediate action  Did “nothing”  Went to pharmacy  Sought medical advice  Other  Missing | 2056 (68)  233 (8)  660 (22)  85 (3)  1 |
| Medical visit  GP visit  Hospital visit | 749 (25)  99 (3) |
| Hospitalization  Median (Q1-Q3) days | 21 (1)  7 (6-11) |
| First medical visit (described n=604)  COVID-19 very likely or probable  COVID-19 not likely or not  Did not talk about COVID-19 | 375 (62)  152 (25)  77 (13) |
| Treatments  Paracetamol  Aspirin  NSAID  Steroids  Antibiotics  Chloroquine, hydroxychloroquine | 1894 (62)  88 (3)  110 (4)  96 (3)  183 (6)  8 (0) |
| Preventive measures following symptoms  Stayed strictly confined  Wore a mask when going outside  Wore a mask in household  Increased hand hygiene  Maintained social distance from household contacts  Household contacts wore face masks  Household contacts surveyed body temperature  Household contacts stayed strictly confined | 1203 (40)  760 (25)  106 (3)  1773 (58)  246 (8)  51 (2)  550 (18)  347 (11) |

*participants from foreign countries and DOM-TOM are not included

Supplementary table 3. Multivariable-adjusted hazard-ratios of COVID-19-Like Symptoms (CLS) according to covariate values – sensitivity analysis 1, defining the CLS as at least one of cough, fever, dyspnea, sudden onset of anosmia, ageusia or dysgeusia of any duration with onset in the last 17 days before the self-administered questionnaire.

|  | Hazard-Ratio* | P-Value |
| --- | --- | --- |
| Age group  <40  [40-50[  [50-60[  [60-70[  >=70 | Reference  0.80 (0.74; 0.87)  0.64 (0.59; 0.70)  0.48 (0.42; 0.55)  0.31 (0.26; 0.37) | <0.0001  <0.0001  <0.0001  <0.0001 |
| Gender  Female  Male | reference  1.02 (0.96; 1.09) | 0.5500 |
| Regions  Ile-de-France  Grand-Est  Other French metropolitan regions | 1.28 (1.19; 1.37)  1.27 (1.16; 1.40)  reference | <0.0001  <0.0001 |
| Living Area  Rural  <20,000 inhab.  20-000-100,000 inhab.  >100,000 inhab. | Reference  1.10 (0.99; 1.22)  1.12 (1.01; 1.24)  1.19 (1.08; 1.30) | 0.0739  0.0353  0.0003 |
| Household size and composition  Nb persons (incl. participant)  1  2  3 or +  Nb children (<18yrs)  0  1 or + | Reference  0.86 (0.79; 0.93)  0.92 (0.83; 1.02)  Reference  1.11 (1.01; 1.22) | 0.0003  0.1231  0.0336 |
| BMI (kg/m2)  <18.5  [18.5; 25[  [25; 30[ (overweight)  >=30 (obese) | 1.04 (0.90; 1.21)  Reference  1.15 (1.08; 1.23)  1.39 (1.27; 1.52) | 0.5516  <0.0001  <0.0001 |
| Professional activity before lockdown  Student  Working  Looking for a job  Retired  Not working due to health conditions  No professional activity (house wife or husband) | 1.21 (0.95; 1.54)  Reference  1.06 (0.92; 1.23)  0.81 (0.71; 0.93)  1.10 (0.85; 1.41)  0.91 (0.75; 1.11) | 0.1198  0.4199  0.0032  0.4783  0.3656 |
| Essential job position  Health care worker (Y vs N)  Other essential job (Y vs N) | 1.09 (0.95; 1.24)  0.98 (0.90; 1.08) | 0.2180  0.7304 |
| Chronic diseases (Y vs N)  Asthma, COPD, other resp. diseases  Anxiety, depression  Other | 1.30 (1.18; 1.44)  1.34 (1.15; 1.55)  1.20 (1.10; 1.328) | <0.0001  0.0001  0.0001 |

* With stratification on source cohort. 6,402 (6%) participants excluded from the multivariate model due to missing values.

Supplementary table 4. Multivariable-adjusted hazard-ratios COVID-19-Like Symptoms (CLS) according to covariate values – sensitivity analysis 2, defining the at-risk period as between March 16, 2020 and the date of the questionnaire for all participants.

|  | Hazard-Ratio* | P-Value |
| --- | --- | --- |
| Age group  <40  [40-50[  [50-60[  [60-70[  >=70 | Reference  0.90 (0.84; 0.97)  0.87 (0.81; 0.95)  0.73 (0.65; 0.82)  0.56 (0.48; 0.65) | 0.0076  0.0009  <0.0001  <0.0001 |
| Gender  Female  Male | reference  0.94 (0.89; 1.00) | 0.0381 |
| Regions  Ile-de-France  Grand-Est  Other French metropolitan regions | 1.39 (1.31; 1.48)  1.41 (1.30; 1.52)  reference | <0.0001  <0.0001 |
| Living Area  Rural  <20,000 inhab.  20-000-100,000 inhab.  >100,000 inhab. | Reference  1.09 (1.00; 1.18)  1.08 (0.99; 1.17)  1.12 (1.04; 1.21) | 0.0501  0.0701  0.0034 |
| Household size and composition  Nb persons (incl. participant)  1  2  3 or +  Nb children (<18yrs)  0  1 or + | Reference  0.97 (0.90; 1.04)  0.98 (0.90; 1.08)  Reference  1.17 (1.07; 1.27) | 0.3998  0.7338  0.0003 |
| BMI (kg/m2)  <18.5  [18.5; 25[  [25; 30[ (overweight)  >=30 (obese) | 1.00 (0.87; 1.14)  Reference  1.13 (1.07; 1.19)  1.27 (1.17; 1.37) | 0.9776  <0.0001  <0.0001 |
| Professional activity before lockdown  Student  Working  Looking for a job  Retired  Not working due to health conditions  No professional activity (house wife or husband) | 0.90 (0.69; 1.18)  Reference  1.06 (0.92; 1.21)  0.87 (0.77; 0.97)  1.07 (0.86; 1.33)  0.87 (0.73; 1.03) | 0.4485  0.4237  0.0151  0.5281  0.1039 |
| Essential job position  Health care worker (Y vs N)  Other essential job (Y vs N) | 1.05 (0.94; 1.18)  0.99 (0.91; 1.07) | 0.4123  0.7794 |
| Chronic diseases (Y vs N)  Asthma, COPD, other resp. diseases  Anxiety, depression  Other | 1.50 (1.39; 1.62)  1.27 (1.13; 1.44)  1.21 (1.12; 1.30) | <0.0001  <0.0001  <0.0001 |

* With stratification on source cohort. 6,861 (6%) participants excluded from the multivariate model due to missing values.
